# Effect of biological sewage sludge and its derived biochar on accumulation of potentially toxic elements by corn (*Zea mays* L.)

**DOI:** 10.1038/s41598-024-56652-8

**Published:** 2024-03-12

**Authors:** Maryam Namdari, Mohsen Soleimani, Nourollah Mirghaffari, Seyyedeh Maryam Kharrazi

**Affiliations:** Department of Natural Resources, Isfahan University of Technology, Isfahan, 84156-83111 Iran

**Keywords:** Soil contamination, Heavy metals, Bioavailability, Risk, Waste management, Biogeochemistry, Environmental sciences

## Abstract

The land application of sewage sludge can cause different environmental problems due to the high content of potentially toxic elements (PTEs). The objective of this study was to compare the effect of urban biological sewage sludge (i.e. the waste of activated sludge process) and its derived biochar as the soil amendments on the bioavailability of PTEs and their bioaccumulation by corn (*Zea mays* L.) under two months of greenhouse conditions. The soil was treated by adding biochar samples at 0 (control), 1, 3, 5% w/w. The diethylenetriamine pentaacetic acid (DTPA)-extractable concentrations of PTEs including Zn, Pb, Cd, Cr, Ni, Fe, and Cu in soil and their accumulation by plant shoot and root were measured. Conversion of the biological sewage sludge into the biochar led to decrease the PTEs bioavailability and consequently decreased their contents in plant tissues. The DTPA extractable metal concentrations of produced biochar in comparison to the biological sewage sludge reduced 75% (Cd), 65% (Cr), 79% (Ni and Pb), 76% (Zn), 91% (Cu) and 88% (Fe). Therefore, the content of Ni, Fe, Zn and Cd in corn shoot was decreased 61, 32, 18 and 17% respectively in application of 5% biochar than of raw sewage sludge. Furthermore, the application of 5% biochar enhanced the physiological parameters of the plants including shoot dry weight (twice) and wet weight (2.25 times), stem diameter (1.70 times), chlorophyll content (1.03 times) in comparison to using 5% raw sewage sludge. The results of the study highlight that application of the biochar derived from urban biological sewage sludge in soil could decrease the risk of PTEs to the plant.

## Introduction

The urban sewage sludge has been used to improve the fertility of agricultural lands. The properties of urban sewage sludge should be improved through the different processes before being used in agricultural lands so that it becomes devoid of hazardous materials^1^.

The non-treated urban sewage can cause the contamination of the environment and has an adverse effect on the quality of the surface and groundwater^2^. The treatment of urban sewage sludge results in the exploitation of the sewage and the retrieval of the used water in addition to environmental preservation^2^. The activated sludge process as a conventional biological aerobic system is broadly used to remove biodegradable compounds from urban and industrial wastewater. However, one of the main defects of the aerobic process (i.e. using bacteria to break down the wastes) is generation of a substantial part (about 50–60%) of waste activated sludge or biological sludge^3^.

The most important ways of waste management and reusing of sewage sludge include land use, agricultural landscapes rehabilitation, sanitary landfill, burning and dumping the sludge in sea^4^. Sewage sludge can be used as a fertilizer in agricultural lands because of possessing organic materials and nutrient content. The organic material of sewage sludge can improve physicochemical properties of soil such as aggregation stability, aeration, water holding capacity, cation exchange capacity and in this way, it improves the growth and performance of agricultural products^4^.

Pyrolysis of biological sewage sludge and production of biochar can be an alternative of dumping and using sludge in agriculture^5,6^. This process can result in decreasing the volume of solid residues (inorganic) and the elimination of the pathogen and organic materials, which is a concern related to the sewage sludge^7^.

Biochar is a solid spongy carbon produced from various carbonaceous materials. It’s made at the temperature of less than 700 °C through the pyrolysis process under an atmosphere lacking or having little oxygen^8^. The solid material obtained from the process contains biomass rich in carbon, aromatic groups, and physical properties suitable for safe and long-lasting storage of carbon in the environment and potentially improvement of the soil quality, plant health and crop yield as well^9,10^.

The primary materials of the biochar can include the garbage and the residue of sawmills, wood chips, sawdust, remains of the urban grass-like lopped grass and branches, roost of poultries, sewage sludge, and waste cardboard materials^11–14^. The biochar can also be produced from the wastes of agricultural products such as wheat straw, corn, rice bran, bagasse, cassava, corncobs^15–18^.

Nowadays the biochar has been introduced as a proper solution in the management of the environment, so there are different ways of producing biochar on different scales in the world^19^. The practical and important uses of biochar which are complementary and are often proposed in environmental management include reducing effects of climate change, remediation and improvement of the soil, waste management, energy production, and its use as an adsorbent^19^.

Thermal treatment through pyrolysis process could be a sustainable alternative for agricultural use of sewage sludge^20^. Comparing to sewage sludge, application of sewage sludge biochar could significantly enhance plants growth and reduced the accumulation of metals in their tissues^21^. Adding the biochar to the soil prevents decreasing soil quality. Recently it has been widely used in modern agriculture, but there are still limitations in using it as a soil fertilizer and the limitations include technical problems, expenses, and the ambiguity in the way its physical and chemical characteristics affect soil fertility^22^.

Biochar also can remediate soil contaminated by potentially toxic elements (PTEs) through mitigating their mobility. The properties of the soil, biochar, and PTEs can primarily affect the success of this process^23^. Hossain et al.^4^ confirmed that the accumulation of PTEs in edible plant tissues was significantly reduced with the application of sewage sludge-derived biochar as a soil amendment^3^. They concluded that the PTEs accumulation rates in the fruits were almost lower than in the sludge-derived biochar treated compared to the treated sewage sludge samples. The application of sewage sludge biochar significantly improved both plant growth and production and decreased the bioavailability and mobility of soil PTEs and thereafter reduced their accumulations in plant tissues^24^. Chagas and Figueiredo^20^ concluded that due to higher pH, P, and K content, pore volume, and specific surface area promotion of sludge biochar compared to sewage sludge, the PTEs bioavailability of biochar was decreased, while the total metal concentration in biochar was increased. Singh et al.^25^ investigated that pyrolysis of sewage sludge and preparation of biochar not only improve soil quality but also reduce the uptake of PTEs, and thereafter application of biochar as a soil amendment has potential benefits in agriculture fields. Ibrahim et al.^26^ acknowledged the reduction of PTEs bioavailability and thus their uptake by plants in contaminated soils with the application of biochar as an environmentally friendly and cost-effective soil amendments. They also observed higher plant dry and wet root and shoot weights^26^. Zhang et al.^27^ also recorded the pivotal role of biochar in the improvements of soil fertility and reduction of PTEs accumulation in plant tissues on account of high porous structure of biochar and its tunable functionality. Nkoh et al.^28^ well stablished the mitigation of the daily uptake of PTEs and hazard quotient and thus their subsequent effects on human health and cancer risk by amending PTEs contaminated soils with biochar.

As mentioned above, due to the presence of various PTEs in the sewage sludge, its use as a fertilizer may increase the risk of HMs bioaccumulation by plants. Therefore, this study was done to analyze the effect of conversion of the biological sewage sludge to biochar on the PTEs bioavailability in the soil and their relevant risk of translocation from soil to plant. This study hypothesizes that the bioavailability of PTEs (including Fe, Zn, Cd, Cr, Cu and Ni) in the soil treated with sewage sludge biochar should be almost lower than biological sludge treated ones. Consequently, the bioconcentration of the PTEs in plant tissues reduces considerably. The study specifically aimed to highlights the benefits of risk mitigation of PTEs accumulation in corn tissues using sewage sludge-derived biochar as a soil amendment.

## Materials and methods

### Sampling and preparing the sewage sludge

The biological sludge was sampled from an urban sewage treatment plant of Chaharmhal-Bakhtiari province (located in the southwest of Iran). In this plant, the treatment was done using the aerobic decomposition and the activated sludge system. The sludge refilled with water was air-dried for 72 h and ground. In order to homogenize the sludge, the grinded samples were sieved to a less than 2 mm particle size. All chemicals used were of analytical grade (Merck, Germany).

### Biochar production

The pyrolysis of prepared sewage sludge was done in a fixed bed reactor at the temperature of 300 °C with an average heating rate of 15 °C/min for 1 h as previously reported^29^. To neutralize the atmosphere, N_2_ was passed through the furnace at the constant flow rate of 1 L/min. The gas was used during the pyrolysis process and also during the cooling time. Finally, the biochar sample was cooled under N_2_ atmosphere, then weighted, and saved in plastic containers. The yield of the biochar production was measured using the ratio of the amount of produced biochar to the amount of primary organic materials^30^.

### Characterization of the raw sewage sludge and the produced biochar

The concentration of PTEs (Zn, Pb, Cd, Cr, Ni, Fe, Cu) was measured according to the standard ASTM D5198-09^31^. The waste sample was mixed with a certain amount of concentrated HNO_3_ and heated for 2 h at 90 to 95 °C. After dissolving of elements, the sample was filtered and the filtrate volume reached to an appropriate volume for subsequent analysis of elements. The bioavailability of PTEs was determined through the diethylene-triaminepentaacetic acid (DTPA) extraction of elements using a 1:2 ratio (w/v) of sample: extract solution (DTPA + CaCl_2_ + triethanolamine (TEA) at pH 7.3) by shaking for 2 h^32^. The amount of the moisture and ashes was measured according to ASTM D2974-20e1^33^. The moisture content was analyzed by drying the sample at 105 °C. Then, the oven-dried sample was heated in a muffle furnace at 600 °C to determine the ash content of the sample. The total Kjeldahl N (through sample digestion with 1:20 w/v sulfuric acid and subsequent distillation), the available P (using Olsen method through extraction with sodium bicarbonate and measured calorimetrically), the exchangeable K (using flame photometer after extraction with ammonium acetate) were measured^34^. According to ASTM D 4980-89, a 10% slurry sample in water was prepared and the pH was measured on the aqueous portion (Research pH meter 3330, Jenway). Electrical conductivity (EC) of the sludge and the biochar samples (1:2.5 solid sample to water) were also measured (Conductivity meter 4310, Jenway)^35^.

### Characterization of soil sample

The soil samples for cultivating plants were taken from the campus of Isfahan University of Technology, Isfahan, Iran at a depth of 0–30 cm. Some characteristics of the used soil such as the soil texture (using hydrometer method), organic material (using Walkley and Black titration method), pH (1:2.5 soil to water), and EC (1:2.5 soil to water) were measured^36^. The total N (Kjeldahl method), the amount of available P (Olsen method), exchangeable K, and CaCO_3_ content were also measured^37^.

The bioavailable concentrations of Zn, Pb, Cd, Cr, Ni, Fe, Cu in soil were determined through sample extraction by DTPA and measured using flame atomic absorption spectrometer (Perkin-Elmer, AAnalyst 700). As evident from the literature, both mobile and potentially mobile species of metals were measured in the DTPA extraction method. It is noteworthy that there was a good correlation between DTPA-extractable metals and elements taken up to plant tissue. Accordingly DTPA extraction is one of the most useful methods for determining the mobile and bioavailable PTEs concentrations in slightly acidic to alkaline soils^24^. Moreover, soil samples were digested using nitric acid for 2 h at 90 to 95 °C to determine total element concentrations and measured by flame atomic absorption spectrometer (Perkin-Elmer AAnalyst 700).

### Plant cultivation

The study was conducted in accordance with guidelines and legislations of Department of Environment and Department of Natural Resources and Watershed Management of Iran to use the plants for cultivation. Accordingly, five seeds of *Zea mays* L. were cultivated in the vase containing 2 kg soil. The raw sewage sludge and the biochar samples were added to the soil separately in four levels of 0 (controls), 1, 3, 5% w/w. The moisture content of the soil was retained according to 50% of the field capacity. Three seedlings having four leaves were kept in each vase and were grown in the greenhouse for 9 weeks. Some plant growth parameters including the thickness of the stem, the number of the leaves and the amount of chlorophyll (Chlorophyll Content Meter, CL-01) were measured.

### Measurement of PTEs plant tissues

To measure the concentration of PTEs in plant tissues, 0.3 g of each dried sample was digested in a thermal block using 7.5 mL concentrated HCl and 2.5 mL concentrated HNO_3_ (Merck, Germany) at 110 °C and after cooling, 1 mL H_2_O_2_ (Merck 30%) was added and heated at 80 °C for an hour and then the volume of sample was reached to 50 mL by adding distilled water^37^. The concentrations of PTEs including Fe, Zn, Ni, Cu, Cr, Pb, and Cd were measured using the flame atomic absorption spectrophotometer (Perkin-Elmer AAnalyst 700). The reference materials of Apple leave 1515 were used for quality control and quality assurance analyses which showed 80 to 93% recovery of the elements.

The bioconcentration factor (BCF) and translocation factor (TF) were calculated to estimate the ability of PTEs to transfer from soil to plant, as below^38^:

$$BCF=\frac{{C}_{root}}{{C}_{Soil}}$$

$$TF=\frac{{C}_{shoot}}{{C}_{root}}$$

where C_root_, C_shoot_, and C_soil_ are each element concentration in the root, shoot, and corresponding soil, respectively.

### Statistical analysis

All the experiments were conducted with three replicates. Data analysis was done using one-way ANOVA analysis and Tukey’s test at the level of 5% by SPSS software.

## Results and discussion

### Characteristics of biochar sample

Biochar production rate at 300 °C was about 80%. As reported previously, the pyrolysis temperature of 300 °C was an efficient factor of biochar production where the mass of biochar decreased with increasing the temperature^6,30^.

Some of the biochar and sewage sludge characteristics are shown in Table 1. The percentage of the ash in the samples enhanced by the increase of temperature. The N content of the biological sewage sludge and the produced biochar samples were 6 and 1.2% w/w, respectively that were less than the amounts reported by the United States Environmental Protection Agency (US-EPA^39^). However, the results showed that the amounts of total N, available phosphorous, and K in the biological sludge were more than the produced biochar which were in accordance with the previous researches^4,40^. The pH and EC of biochar samples were lower than that of raw materials. The increase of biochar pH in comparison to the raw materials is usually observed in the biochar resulted from the sewage sludge and the other organic materials^41–43^. This process has a relationship with the effect of the decrease in the acid groups during the heating process on dehydrating of the organic materials. Furthermore, the increase of pH can happen because of the aggregation of the organic materials in the biochar which may be a result of the separation of the salt of alkaline elements (e.g. Na and K) from the organic matrix and formation of carbonates (MgCO_3_ and CaCO_3_) with the increase of temperature^40–44^. However, it’s expected to have acidic biochar in low temperatures^45^, as it’s seen (slightly acidic) for the produced biochar sample.

**Table 1:** Characteristics of the biological sewage sludge and produced biochar.

Parameter	Unit	Sewage sludge	Biochar
Moisture	%	8.0	2.0
Ash	%^a^	23.7 ± 0.42	34.4 ± 0.57
Total N	%	3.26 ± 0.24	1.73 ± 0.35
Available P	mg/kg	3874 ± 98	1298 ± 70
Exchangeable K	mg/kg	3293 ± 114	1400 ± 39
Total Na	mg/kg	856 ± 134	1054 ± 45
pH	–	7.1 ± 0.2	6.7 ± 0.1
EC	dS/m	3.38 ± 0.04	1.76 ± 0.04

^a^On dry basis.

### Characterization of the soil sample

Some chemical and physical characteristics of soil sample are presented in Table 2. The results pointed out that the soil texture was sandy loam, alkaline, none-salty with a high amount of CaCO_3_. Besides, the P, K and organic contents of the soil were low which could adversely affect the plant growth. However, it could effectively show the results of amendments on the plant growth. Additionally, the soil was not saline and could not adversely affect plant growth, but had low organic materials (less than 2%) which was usual for most soils in arid environments and might have some limitations for the soil physicochemical quality.

**Table 2:** Characteristics of the soil sample.

Parameter	Unit	Studied soil
Soil texture	–	Sandy loam
Total N	%	1.157 ± 0.14
Available P	mg/kg	ND
Exchangeable K	mg/kg	374 ± 10
Organic matter	%	1.227 ± 0.100
CaCO_3_	%	29.5 ± 0.5
pH	–	8.3 ± 0.1
EC	dS/m	0.04 ± 0.001

### Analysis of PTEs in biological sewage sludge and the produced biochar

The total concentrations of PTEs including Cd, Cr, Ni, Pb, Zn, Cu, and Fe in the biological sewage sludge and the produced biochar are shown in Table 3. The results showed that the concentrations of all the PTEs except Ni in the biochar sample were lower than the critical level according to the US-EPA^46^ and California Food and Agriculture Organization^47^. In the other hand, the total contents of PTEs in biochar samples were much higher than of raw sewage sludge (2.9 to 11.7 times more). However, the concentration of bioavailable PTEs (DTPA extractable) was significantly reduced in biochar samples in comparison to the raw materials (Table 4). It was about 2 times less for Cd, 1.6 times less for Ni and Pb, 1.4 times less for Zn and Fe, and 1.3 times less for Cu. Although the concentrations of the PTEs increased in the pyrolysis process, the results of the DTPA- extraction analysis showed that the pyrolysis process significantly decreased the bioavailability of these elements (1.3 to 2 times) in the compound structure. It was more efficient for Cd which is also more toxic than the other PTEs. This is an important feature of the biochar to be used in the soil. Therefore, using the biochar as a soil amendment could be safer than sewage sludge because of the low bioavailability of the PTEs and the improvement of soil quality and fertility, in different soils^8^. Chagas and Figueiredo^20^ concluded that despite increasing total PTEs concentration in the biochar compared to the sewage sludge, their bioavailability was decreased, which could be attributed to higher pH, P, and K content and pore volume, as well as specific surface area promotion. The observed results were also supported by previous researches showing the increase of PTEs content in biochar samples compared to raw materials, whereas the DTPA-extractable (i.e. bioavailable form) of PTEs in biochar was significantly reduced^24,48^.

**Table 3:** Total PTEs concentration of the biological sewage sludge and produced biochar (mg/kg).

Metals	Sewage sludge	Biochar	Standard of U.S. EPA (1993)	Standard of California food and agriculture organization
Cd	1.2 ± 0.1	2.4 ± 0.1	85	1–15
Cr	43.26 ± 3.74	54.19 ± 3.38	3000	0.75–83
Ni	73.81 ± 7.76	119.99 ± 9.92	75	32–43
Pb	55.03 ± 8.90	89.46 ± 6.15	420	2.5–110
Zn	728.33 ± 57.24	1048.33 ± 11.81	7500	ND
Cu	128.03 ± 28.98	160.51 ± 4.83	4300	4.5–330
Fe	5330.83 ± 583.50	7516.67 ± 638.52	–	–

**Table 4:** The DTPA extractable concentrations of PTEs of biological sewage sludge and produced biochar (mg/kg).

Metal	Sewage sludge	Biochar
Cd	0.3 ± 0.1	0.1 ± 0.0
Cr	0.62 ± 0.06	0.15 ± 0.00
Ni	3.21 ± 0.57	0.67 ± 0.27
Pb	13.46 ± 0.48	2.92 ± 0.38
Zn	316.40 ± 21.78	38.75 ± 3.11
Cu	17.01 ± 0.14	1.45 ± 0.43
Fe	187 ± 39.60	63.92 ± 8.95

### Effect of biological sewage sludge and biochar on plant growth parameters

**Figure 1:**
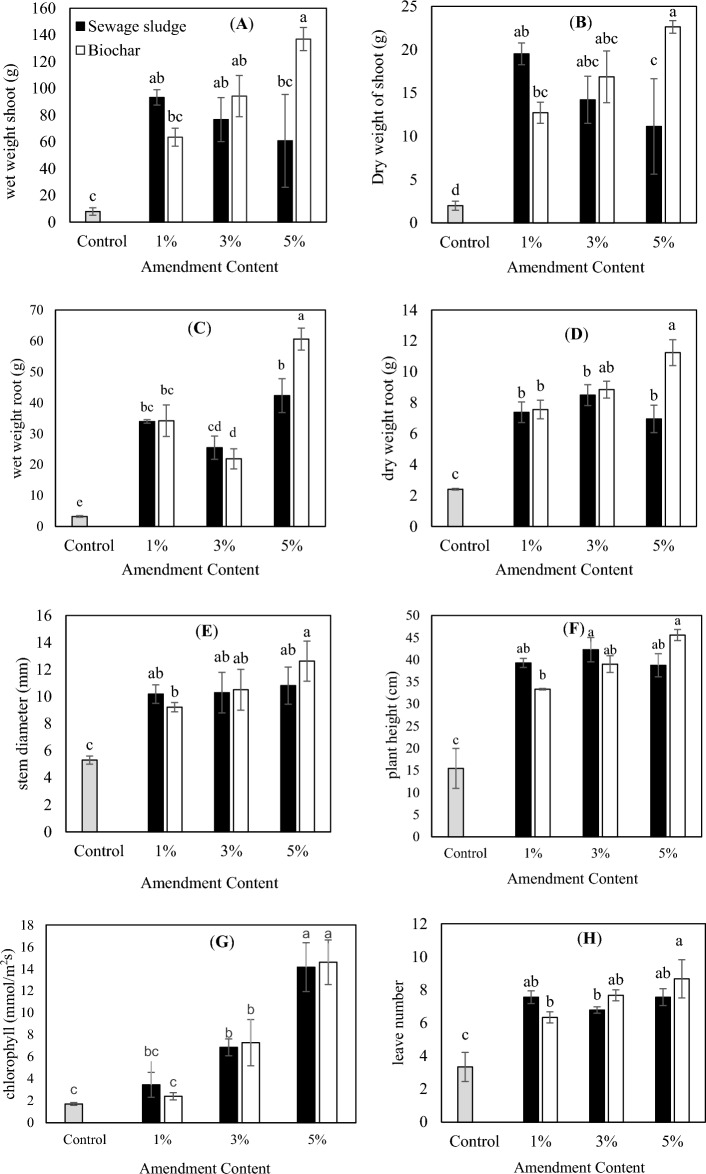
Effect of application of different amounts of biological sewage sludge and biochar on the growth parameters of *Zea mays* L. (**A**) wet weight shoot, (**B**) dry weight shoot, (**C**) wet weight root, (**D**) dry weight root, (**E**) stem diameter, (**F**) plant height, (**G**) chlorophyll and (**H**) leave number. The same letters show the insignificant difference by the Tukey test (*P* > 0.05).

**Figure 2:**
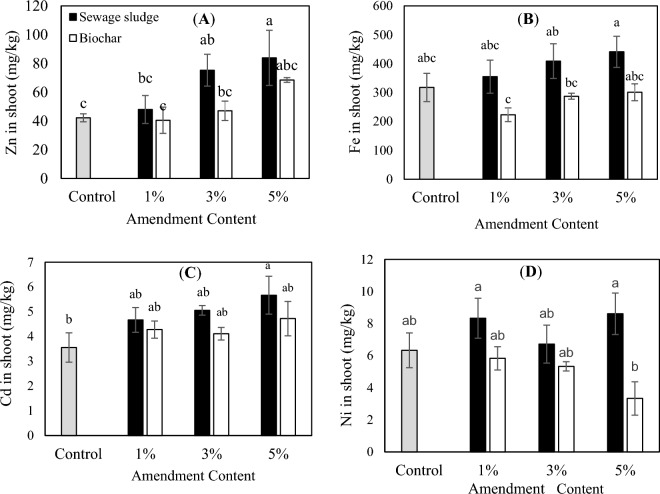
Effect of application of different amounts of biological sewage sludge and biochar on the PTEs shoot contents of *Zea mays* L. (**A**) Zn, (**B**) Fe, (**C**) Cd, and (**D**) Ni. The same letters show the insignificant difference by the Tukey test (*P* > 0.05).

The root and shoots weight of the corn plants after being harvested are seen in Figs. 1 and 2, respectively. There was a significant difference between the treatments and the controls in terms of the effect on the dry weight of the root and shoot of the plant. The application of biological sewage sludge and produced biochar as soil amendments increased the weight of the root and shoot of the corn plant. Application of biochar modified soil physical and chemical properties and concequently improved plant yield and quality^49^. This could be ascribed to the high level of nutrients in the used amendments, improvement of soil aeration and its water holding capacity, and therefore supporting good environmental conditions for plants^4^.

The maximum weight of the plant root and shoot was related to the biochar and the minimum one was related to the control. The same results were obtained for summer squash by application of biochar^26^. Meanwhile, soil fertility in biochar treated soil could be modified due to biochar tunable functionality and high porous structure^27^. The growth improvement of plant concerning the application of biochar depends not only on the ingredients of the primary organic material enriched in the biochar during the pyrolysis process but also on the effect of the biochar on the physical, chemical, and biological properties of the soil^50^. The increase of plant yield as a result of the using biochar and fertilizers can be attributed to creating the optimal conditions for the plants (e.g. corn, garlic, bean and cherry tomato) to grow and better uptake of water and other essential elements^4,51–55^. Similar observations were made by Leiva‐Suárez et al.^56^ by using the sewage sludge‐derived char as a soil amendment. They concluded that the produced char provided N and P for *Lolium perenne* growth during a second cropping period^56^.

Figure 1 represents the effect of different treatments on the amount of chlorophyll of the plant. There was a significant difference between the amount of chlorophyll of the treatments and control. The results showed that the amount of chlorophyll increased with the increase of the added biological sludge and biochar into the soil. Since the chlorophyll content could be an appropriate index to show the photosynthesis and plant productions, healthy plants in the presence of biochar samples were expected to grow further and consist of more chlorophyll^57^. The other plant growth parameters including stem diameter and leaf number also revealed the positive effect of using biochar as a soil amendment as reported before^54^.

### Effect of biological sewage sludge and biochar on metal accumulation by plants

**Figure 3:**
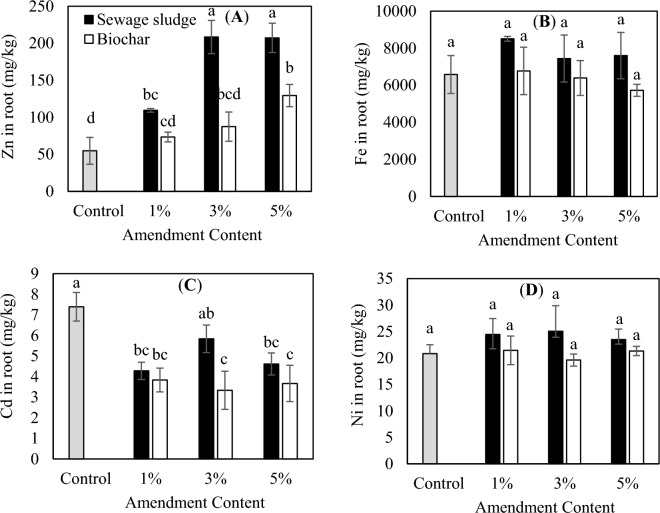
Effect of application of different amounts of biological sewage sludge and biochar on the PTEs root contents of *Zea mays* L. (**A**) Zn, (**B**) Fe, (**C**) Cd, and (**D**) Ni. The same letters show the insignificant difference by the Tukey test (*P* > 0.05).

The concentrations of Zn, Fe, Ni, and Cd in the shoots were much lower than the roots (Figs. 2 and 3). This implies that *Zea mays* L. roots prevent the translocation of PTEs and protect its edible parts from severe contamination. Similar observations were made by other researchers while studying the metal accumulation by different plant species^38,40,58^. It can be assumed that the main accumulation receptors for PTEs in the plant species are plant roots^4,38^. The Fe concentration in the shoots showed the highest value among all metals, followed by Zn, Ni, and Cd; the same trend was observed for the plant roots (Figs. 2 and 3).

The results indicated that the maximum amount of Zn in the shoots was related to the treatment of the sludge (Fig. 2a). The reason can be the lower extent of bioavailability of the metal in the biochar in comparison with the biological sewage sludge. Also, the concentration of Zn increased in the shoots with the increase of the level of added biochar and sludge. The maximum root concentration of Zn in the treatments of the sludge was corresponded to the level of 3 and 5%. Besides, the amount of the Zn concentration in roots enhanced with the increase of the added sludge and biochar to the soil. Based on the literature, Zn had a high internal translocation capacity in comparison with other studied metals^38^. There was not a significant difference (*P* > 0.05) in Ni, Fe, and Cd accumulation by plant root and shoot in all treatments (Figs. 2 and 3). The reasons for insignificant differences between these metals in comparison with control could be related to a possible interaction of the elements in the treated soils with the other soil properties. Yue et al.^24^ also concluded that biochar amendment alters the fractions of soil PTEs which depends on the used biochar, type of element, and soil conditions. It is noteworthy that the lowest concentrations of Fe, Ni, and Cd were observed in biochar treated samples, especially in the treatment of 5% biochar compared with biological sewage sludge ones. In this sense, the highest concentration of Cd in the roots was observed in 5% sludge treated sample and its lowest concentration was in the shoots of 3 and 5% biochar treated samples. As evident from literature, due to the high mobility and bioavailability of Cd to plants, it could be a major element with a high health risk to humans^38,40^. Therefore, reducing its bioavailability, particularly in contaminated soils, play a decisive role in human health protection. Yue et al.^24^ confirmed that the application of sewage sludge biochar not only improved plant growth and therefore plant production, but also decreased the bioavailability and mobility of soil PTEs and thereafter reduced their accumulations in plant tissues. In good agreement with this study, Hossain et al.^4^ revealed that the accumulation of Cd, Cu, Zn, Cr, As, and Pb in edible tissues of the studied plant was inhibited with the application of sewage sludge biochar as a soil amendment. They concluded that in comparison to the wastewater sludge treated sample, the PTEs accumulation rates in the fruits were almost lower than in the sludge biochar treated sample.

The BCF of Zn, Fe, Cd, and Ni in *Zea mays* L., and the concentration of PTEs in the roots to those in the soil, are shown in Table 5. The results demonstrated that *Zea mays* L. root possessed different PTEs accumulation capacities compared to other plants (e.g. wheat)^38^. The BCF of samples was in the order of Cd > Zn > Ni > Fe, implying that Cd showed the higher transfer factor from treated soil to roots than those of Zn, Ni, and Fe. The presence of Cd in exchangeable and carbonate form in the soil, renders bioavailable for plant roots easily. In alkaline soil, the different complex of Cd ions (CdCl^+^, CdOH^+^, CdCl_3_^−^, and CdCl_4_^−2^) are more abundant which are generally more available to roots, leads to higher BCF for Cd^59^. The BCF in biochar treated samples were much lower than those in sewage sludge treated samples (Table 5). This can be explained by reduction of PTEs bioavailability due to the application of biochar and consequently lower BCF values. Similar observations were made by previous researches which confirmed the reduction in the bioavailability of metals in biochar amended soils^4,21,24,25^. Table 6 showed the TF of PTEs in corn cultivated in sewage sludge and biochar treated soil. The transfer of PTEs from the root of *Zea mays* L. to its shoot was in the order of Cd > Zn > Ni > Fe, which is the same order established for BCF. The TF values for all PTEs were  > 1, except Cd, indicating that corn tissues had a weak transfer capability of these elements from roots to shoots and they mostly accumulated in the roots.

**Table 5:** Bioconcentration factor (BCF) for Zn, Fe, Cd, and Ni in *Zea mays* L. under different treatments.

Metal	Amendment content			
		1%	3%	5%
Zn	Sewage sludge	0.665	1.045	1.154
	Biochar	0.321	0.411	0.491
Fe	Sewage sludge	0.161	0.134	0.132
	Biochar	0.114	0.108	0.096
Cd	Sewage sludge	1.624	2.482	1.718
	Biochar	0.909	1.176	0.777
Ni	Sewage sludge	0.362	0.398	0.372
	Biochar	0.316	0.286	0.328

**Table 6:** Translocation factor (TF) for Zn, Fe, Cd, and Ni in *Zea mays* L. under different treatments.

Metal	Amendment content			
		1%	3%	5%
Zn	Sewage sludge	0.438	0.361	0.405
	Biochar	0.550	0.538	0.529
Fe	Sewage sludge	0.042	0.055	0.058
	Biochar	0.033	0.045	0.053
Cd	Sewage sludge	1.091	0.867	1.229
	Biochar	1.116	1.233	1.288
Ni	Sewage sludge	0.341	0.268	0.366
	Biochar	0.272	0.272	0.156

The result revealed that the application of biochar as a soil amendment reduces the bioavailability of PTEs and therefore reduce the Zn, Fe, Ni and Cd accumulations in the plant tissues in comparison to the application of raw sludge. Biochar can stabilize contaminants in soil through some mechanisms like altering soil chemistry and exchanging ions and physical entrapping on their surfaces. Depends on the PTEs functionality, positive surface biochars can stabilize anionic contaminants while negative surface biochars have a large capacity to interact with cationic contaminants and thus reduce their bioavailability^30^. The results were well consistent with Fathianpour et al.^60^ who reported remarkable stabilization of PTEs in soil using biochar. They also deduced the longer the time of biochar in contaminated soil, the more elements were stabilized. Pandey et al.^61^ acknowledged the low mobility of Cr, Cd, Cu, Pb, Ni, Zn, Mg and Fe contents in soils and therefore lower PTEs uptake rates in biochar application, compared with the control treatments. Ibrahim et al.^26^ also concluded a significant reduction in BCF and TF of the PTEs and therefore their concentration in root and shoot on account of the application of biochar in soil and reductions in their bioavailability.

### Effect of biological sewage sludge and biochar on soil metal concentration

**Figure 4:**
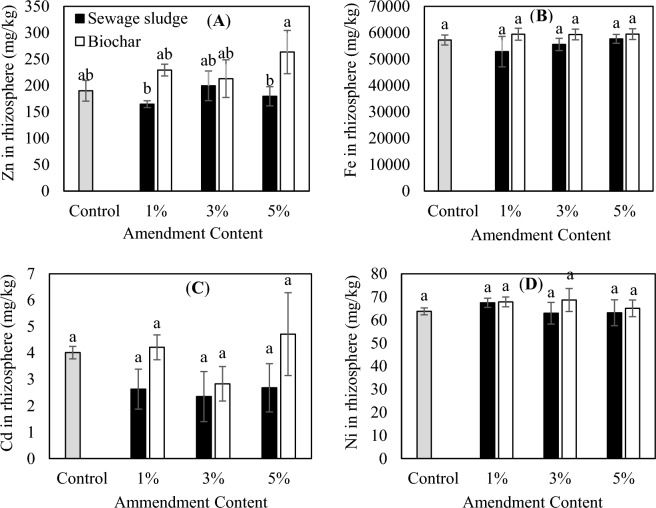
Effect of application of different amounts of biological sewage sludge and biochar on the PTEs concentrations of rhizosphere. (**A**) Zn, (**B**) Fe, (**C**) Cd, and (**D**) Ni. The same letters show the insignificant difference by the Tukey test (*P* > 0.05).

Figure 4 shows the concentration of Zn, Fe, Ni, and Cd in the soil treated by the biological sludge and the biochar samples. The maximum metal concentration was related to the treatment of the biochar 5% but there wasn’t a significant difference (*P* > 0.05) between the treatments and control in the soil metal concentrations. (Fig. 4). Probably the reason was the more stability and less bioavailability of the PTEs in the biochar in comparison with the sludge^62^. It could be concluded that the application of biochar amendment might influence the fractions of PTEs in soil in three possible different mechanisms, precipitation, adsorption, and biological transformation. The presence of carbonates, phosphates, and oxides (inorganic components) could affect relative fractions of PTEs in the biochar-treated soil through precipitation. Adsorption of PTEs through coordination, chelation might be conducted due to the presence of diverse functional groups such as hydroxyl, carboxyl, and phenolic. Microbial decomposition of biochar could reduce oxidizable PTEs and alter some element valence^24^. According to the literature, the majority of metals in amended soils were in residual fraction and were less bioavailable even after 3 years^63^. It is noteworthy that the accumulation of metals in soil and their bioavailability and also their uptake to plant tissues were depending not only on the chemistry and mobility of the metals in the amendment but also on the physicochemical properties of the soil and also the type of plant species^3^.

## Conclusion

The pyrolysis of the wastes such as the sewage sludge not only provides proper waste management but also because of the useful chemical properties could remediate contaminated soils and might be used as a soil amendment. In this study, the effect of raw biological sewage sludge from an urban wastewater treatment and it’s derived biochar on PTEs accumulation by corn was investigated. Although total contents of PTEs in biochar samples were 1.3 to 2 times more that the raw materials, the bioavailable contents (i.e. DTPA extractable fractions) were much lower (2.9 to 11.7 times) than of the raw materials. It revealed the significant effect of conversion of the sludge to biochar in reduction of PTEs accumulation by the plant and consequently decreasing their relevant risks. This was proved by higher growth parameters (e.g. dry weight and stem diameter) of the plants grown in biochar amended soils in comparison to those planted in soils which obtained raw sewage sludge. It means that conversion of the biological sewage sludge into biochar can reduce the risk of metal transfer to the plant and decreases the bioconcentration factor because of reducing bioavailability of PTEs and on the other hand, can improve growth of the plants. It could be also an efficient approach for control of pathogens associated with raw sewage sludge which however needs more investigation.

## Data Availability

All data generated or analyzed during this study are included in this published article.

## References

[1] Tomczyk B, Siatecka A, Jędruchniewicz K, Sochacka A, Bogusz A, Oleszczuk P (2020). Polycyclic aromatic hydrocarbons (PAHs) persistence, bioavailability and toxicity in sewage sludge- or sewage sludge-derived biochar-amended soil. Sci. Total Environ..

[2] Tchobanoglous G, Burton FL, Stensel HD, Metcalf, Eddy I (2003). Wastewater Engineering: Treatment and Reuse.

[3] Barrios JA, Cano A, Rivera FF, Cisneros ME, Durán U (2021). Efficiency of integrated electrooxidation and anaerobic digestion of waste activated sludge. Biotechnol. Biofuel..

[4] Hossain MK, Strezov V, Nelson PF (2015). Comparative assessment of the effect of wastewater sludge biochar on growth, yield and metal bioaccumulation of cherry tomato. Pedosphere.

[5] Hwang IH, Ouchi Y, Matsuto T (2007). Characteristics of leachate from pyrolysis residue of sewage sludge. Chemosphere.

[6] Tarelho LAC, Hauschild T, Vilas-Boas ACM, Silva DFR, Matos MAA (2020). Biochar from pyrolysis of biological sludge from wastewater treatment. Energy Rep..

[7] Caballero JA, Front R, Marcilla A, Conesa JA (1997). Characterization of sewage sludges by primary and secondary pyrolysis. J. Anal. Appl. Pyrolysis.

[8] Blackwell, P., Reithmuller, G. & Collins, M. *Biochar Applications to Soil*. Vol. 2. Biochar for Environmental Management: Science and Technology, (2009).

[9] Shackley, S. *et al.* in *Geoengineering Responses to Climate Change: Selected Entries from the Encyclopedia of Sustainability Science and Technology* (Eds. Tim Lenton & Naomi Vaughan) 73–140 (Springer, 2013).

[10] Xue Y (2012). Hydrogen peroxide modification enhances the ability of biochar (hydrochar) produced from hydrothermal carbonization of peanut hull to remove aqueous heavy metals: Batch and column tests. Chem. Engin. J..

[11] Beesley L, Moreno-Jiménez E, Gomez-Eyles JL, Harris E, Robinson B, Sizmur T (2011). A review of biochars’ potential role in the remediation, revegetation and restoration of contaminated soils. Environ. Pollut..

[12] Hazrati S, Farahbakhsh M, Cerdà A, Heydarpoor G (2021). Functionalization of ultrasound enhanced sewage sludge-derived biochar: Physicochemical improvement and its effects on soil enzyme activities and heavy metals availability. Chemosphere.

[13] Kharrazi SM, Soleimani M, Jokar M, Richards T, Pettersson A, Mirghaffari N (2021). Pretreatment of lignocellulosic waste as a precursor for synthesis of high porous activated carbon and its application for Pb (II) and Cr (VI) adsorption from aqueous solutions. Int. J. Biol. Sci..

[14] Koide RT, Petprakob K, Peoples M (2011). Quantitative analysis of biochar in field soil. Soil Biol. Biochem..

[15] Rodriguez JA, Lustosa Filho JF, Melo LCA, de Assis IR, de Oliveira TS (2020). Influence of pyrolysis temperature and feedstock on the properties of biochars produced from agricultural and industrial wastes. J. Anal. Appl. Pyrolysis.

[16] Wijitkosum S, Jiwnok P (2019). Elemental composition of biochar obtained from agricultural waste for soil amendment and carbon sequestration. Appl. Sci..

[17] Zavalloni C (2011). Microbial mineralization of biochar and wheat straw mixture in soil: A short-term study. Appl. Soil Ecol..

[18] Gonzaga MIS, Mackowiak C, de Almeida AQ, de Carvalho Junior JIT, Andrade KR (2018). Positive and negative effects of biochar from coconut husks, orange bagasse and pine wood chips on maize (*Zea mays* L.) growth and nutrition. Catena.

[19] Lehmann, J. & Joseph, S. *Biochar Environ. Manage*. 1st ed., 404 Earthscan (2009).

[20] Chagas JKM, Figueiredo CC (2021). Long-term effects of sewage sludge-derived biochar on the accumulation and availability of trace elements in a tropical soil. J. Environ. Qual..

[21] Zhang J (2021). Land application of sewage sludge biochar: Assessments of soil-plant-human health risks from potentially toxic metals. Sci. Total Environ..

[22] Ladygina N, Rineau F (2013). Biochar and Soil Biota.

[23] Palansooriya KN (2022). Prediction of soil heavy metal immobilization by biochar using machine learning. Environ. Sci. Technol..

[24] Yue Y, Cui L, Lin Q, Li G, Zhao X (2017). Efficiency of sewage sludge biochar in improving urban soil properties and promoting grass growth. Chemosphere.

[25] Singh S (2020). A sustainable paradigm of sewage sludge biochar: Valorization, opportunities, challenges and future prospects. J. Clean. Prod..

[26] Ibrahim EA, El-Sherbini MAA, Selim EMM (2022). Effects of biochar on soil properties, heavy metal availability and uptake, and growth of summer squash grown in metal-contaminated soil. Sci. Hortic..

[27] Zhang R-H (2022). Effects of biochar on berseem clover (*Trifolium alexandrinum*, L.) growth and heavy metal (Cd, Cr, Cu, Ni, Pb, and Zn) accumulation. Chemosphere.

[28] Nkoh JN (2022). Reduction of heavy metal uptake from polluted soils and associated health risks through biochar amendment: A critical synthesis. J. Hazard. Mater. Adv..

[29] Jazini R, Soleimani M, Mirghaffari N (2018). Characterization of barley straw biochar produced in various temperatures and its effect on lead and cadmium removal from aqueous solutions. Water Environ. J..

[30] Agrafioti E, Bouras G, Kalderis D, Diamadopoulos E (2013). Biochar production by sewage sludge pyrolysis. J. Anal. Appl. Pyrolysis.

[31] ASTM D5198-09, *Standard Practice for Nitric Acid Digestion of Solid Waste*, https://webstore.ansi.org/standards/astm/astmd519809red (2024).

[32] Lindsay WL, Norvell WA (1978). Development of a DTPA soil test for zinc, iron, manganese, and copper. Soil Sci. Soc. Am. J..

[33] ASTM D2974-20e1, *Standard Test Methods for Determining the Water (Moisture) Content, Ash Content, and Organic Material of Peat and Other Organic Soils*. https://www.astm.org/d2974-20e01.html (2020).

[34] Page, A. L. *Methods of Soil Analysis, Part 2: Microbiological and Biochemical Properties*. 1159. American Society of Agronomy, Soil Science Society of America (1982).

[35] Cheng CH, Lehmann J, Thies JE, Burton SD, Engelhard MH (2006). Oxidation of black carbon by biotic and abiotic processes. Organ. Org. Geochem..

[36] Sparks DL (1996). Methods of Soil Analysis: Part 3 Chemical Methods.

[37] Lomonte C, Gregory D, Baker AJM, Kolev SD (2008). Comparative study of hotplate wet digestion methods for the determination of mercury in biosolids. Chemosphere.

[38] Rezapour S, Atashpaz B, Moghaddam SS, Damalas CA (2019). Heavy metal bioavailability and accumulation in winter wheat (*Triticum aestivum* L.) irrigated with treated wastewater in calcareous soils. Sci. Total Environ..

[39] US-EPA, *Clean Water Act Section 503*. **58**, 124 (1993).

[40] Chen H, Yuan X, Li T, Hu S, Ji J, Wang C (2016). Characteristics of heavy metal transfer and their influencing factors in different soil–crop systems of the industrialization region. China. Ecotoxicol. Environ. Saf..

[41] Guo K (2020). Pyrolysis temperature of biochar affects ecoenzymatic stoichiometry and microbial nutrient-use efficiency in a bamboo forest soil. Geoderma.

[42] Luo Y (2018). Pyrolysis temperature during biochar production alters its subsequent utilization by microorganisms in an acid arable soil. Land Degrad. Dev..

[43] Zhao S-X, Ta N, Wang X-D (2017). Effect of temperature on the structural and physicochemical properties of biochar with apple tree branches as feedstock material. Energies.

[44] Zielińska A, Oleszczuk P, Charmas B, Skubiszewska-Zięba J, Pasieczna-Patkowska S (2015). Effect of sewage sludge properties on the biochar characteristic. J. Anal. Appl. Pyrolysis.

[45] Hossain MK, Strezov V, Chan KY, Ziolkowski A, Nelson PF (2011). Influence of pyrolysis temperature on production and nutrient properties of wastewater sludge biochar. J. Environ. Manag..

[46] US-EPA, *Background Report on Fertilizer Use, Contaminants and Regulations*. 406 Office of Pollution Prevention and Toxics, (1999).

[47] California Department of Food and Agriculture, *Evaluation of Heavy Metals & Dioxin in Inorganic Commercial Fertilizers and California Cropland Soils*. Metals Report 12-23-04 (2004).

[48] Wan Y (2020). Accumulation and bioavailability of heavy metals in an acid soil and their uptake by paddy rice under continuous application of chicken and swine manure. J. Hazards. Mater..

[49] Liang M, Lu L, He H, Li J, Zhu Z, Zhu Y (2021). Applications of biochar and modified biochar in heavy metal contaminated soil: A descriptive review. Sustainability.

[50] Major J, Rondon M, Molina D, Riha SJ, Lehmann J (2010). Maize yield and nutrition during 4 years after biochar application to a Colombian savanna oxisol. Plant Soil.

[51] Rodda MRC (2006). Estímulo no crescimento e na hidrólise de ATP em raízes de alface tratadas com humatos de vermicomposto: I - efeito da concentração. Rev. Bras. Cienc. Solo..

[52] Crane-Droesch A, Abiggail C (2012). Biochar Increases Maize Yields and Smalholder Profitability.

[53] Marinari S, Masciandaro G, Ceccanti B, Grego S (2000). Influence of organic and mineral fertilisers on soil biological and physical properties. Bioresour. Technol..

[54] Song XD, Xue XY, Chen DZ, He PJ, Dai XH (2014). Application of biochar from sewage sludge to plant cultivation: Influence of pyrolysis temperature and biochar-to-soil ratio on yield and heavy metal accumulation. Chemosphere.

[55] Prapagdee S, Tawinteung N (2017). Effects of biochar on enhanced nutrient use efficiency of green bean, *Vigna radiata* L.. Environ. Sci. Pollut. Res..

[56] Leiva-Suárez B, Paneque M, Rosa JMDI, González-Pérez JA, Leiva MJ, Knicker H (2021). Soil amendment with sewage sludge-derived chars increases C-sequestration potential and provides N and P for plant growth during a second cropping period with Lolium perenne. Eur. J. Soil Sci..

[57] Krugh B, Bickham L, Miles D (1994). The solid-state chlorophyll meter: A novel instrument for rapidly and accurately determining the chlorophyll concentrations in seedling leaves. Maize Genet. Coop. News Lett..

[58] Xiao-Rui W, Sheng-Lu Z, Shao-Hua W (2016). Accumulation of heavy metals in different parts of wheat plant from the Yangtze river delta, China. Int. J. Agric. Biol..

[59] Huang R (2020). Evaluation of phytoremediation potential of five Cd (hyper)accumulators in two Cd contaminated soils. Sci. Total Environ..

[60] Fathianpour A, Taheriyoun M, Soleimani M (2018). Lead and zinc stabilization of soil using sewage sludge biochar: Optimization through response surface methodology. Clean Soil Air Water.

[61] Pandey B, Suthar S, Chand N (2022). Effect of biochar amendment on metal mobility, phytotoxicity, soil enzymes, and metal-uptakes by wheat (*Triticum aestivum*) in contaminated soils. Chemosphere.

[62] Gascó G, Paz-Ferreiro J, Méndez A (2012). Thermal analysis of soil amended with sewage sludge and biochar from sewage sludge pyrolysis. J. Therm. Anal. Calorim..

[63] Hamidpour M, Afyuni M, Khadivi E, Zorpas A, Inglezakis V (2012). Composted municipal waste effect on chosen properties of calcareous soil. Int. Agrophys..

